# Nurse-Led Brief Intervention for Enhancing Safe Sex Practice Among Emerging Adults in Hong Kong Using Instant Messaging: Feasibility Study

**DOI:** 10.2196/52695

**Published:** 2024-03-20

**Authors:** Sharon Hoi Lam Pak, Man Ping Wang, Anne M Teitelman, Janet Yuen Ha Wong, Daniel Yee Tak Fong, Edmond Pui Hang Choi

**Affiliations:** 1 School of Nursing LKS Faculty of Medicine The University of Hong Kong Hong Kong China (Hong Kong); 2 School of Nursing University of Pennsylvania Philadelphia, PA United States; 3 College of Nursing Thomas Jefferson University Philadelphia, PA United States; 4 School of Nursing and Health Studies Hong Kong Metropolitan University Hong Kong China (Hong Kong)

**Keywords:** condom use, emerging adults, HIV prevention, IM intervention, mHealth, nurse-led intervention, safer sex practice, sexual health, sexually transmitted infections, text-messaging

## Abstract

**Background:**

The incidence of sexually transmitted infections has been increasing throughout the world. Additionally, substantial changes in emerging adults’ attitudes toward sex and the popularization of premarital sex could further affect the diagnosis and treatment of sexually transmitted infections. With the high acceptability and effectiveness of instant messaging (IM) interventions for health promotion, there is potential for such interventions to improve condom use knowledge and promote safer sex practice.

**Objective:**

The study evaluates the feasibility of a nurse-led IM intervention to promote safer sex practices in emerging adults.

**Methods:**

A 30-minute adaptive IM intervention and a 5-day booster dose of daily messages after 2 weeks through WhatsApp (Meta Platforms, Inc) were conducted with emerging adults in local universities in Hong Kong aged between 18 and 29 years with previous sexual experience. A questionnaire was distributed 1 week after the intervention that measured the consistency in condom use, the change in condom use knowledge and attitudes, and the acceptability of the intervention. The feasibility of the intervention was assessed by Bowen’s feasibility framework.

**Results:**

A total of 20 participants completed the intervention and questionnaire. Results showed (1) high satisfaction level (mean satisfaction score: 9.10/10), (2) high demand of the intervention (retention rate: 95%), (3) smooth implementation of the intervention, (4) high practicality (13/20, 65% of the participants viewed IM to be an effective means of intervention), (5) potential integration of the intervention, and (6) significant mean increase in condom use knowledge and attitudes (mean increase 9.05; *t_19_*=3.727; 95% CI 3.97-14.13; *P*=.001).

**Conclusions:**

The IM intervention was feasible, acceptable, and had potential impacts on improving safer sex practices. These findings will support the future development of IM interventions in the arena of sexual health promotion.

## Introduction

Reducing sexually transmitted infections (STIs) has been identified as a global health sector strategy by the World Health Organization (WHO) [1]. The WHO reported that there are more than 1 million STIs acquired every day and that estimated that there are around 374 million new cases each year [2]. Globally, almost 57 million disability‐adjusted life years are lost to STIs [2]. Among all STIs, human papillomavirus (HPV) is the most common infection. In severe cases, this will further lead to invasive cervical cancer [3].

In Hong Kong, there was nearly a 3-fold increase in the incidence of diagnosed HIV from 250 cases in 2011 to 725 cases in 2015 [4]. After reaching its peak in 2015, the number of cases gradually decreased to 505 in 2020. A sharp decline in STI cases was noted from 2020 to the third quarter of 2021. However, the decrease could be due to reduced screening or testing during the COVID-19 pandemic. Although no specific data were found in the context of Hong Kong, studies in the United States and Europe showed a relationship between COVID-19 cases and STI-reported cases. Results reflected that the reported cases were much lower than the expected cases in both countries [5,6]. Hence, the situation is still alarming, as the pandemic could have concealed the real size of the population with STI infection.

In addition, the asymptomatic nature of STIs and a substantial change in Hong Kong people’s attitudes toward sex may further delay the diagnosis and treatment of STIs [7]. For instance, premarital sex and high-risk sexual behaviors are becoming more common. According to the Youth Sexuality Study by the Family Planning Association of Hong Kong, the proportion of youth practicing safe sex (use of condoms during sexual intercourse) decreased from 1996 to 2011 [8]. Among male respondents, the number decreased significantly, from 66.4% in 1996 to 21.5% in 2011, while among female respondents, the number decreased gradually from 55.2% in 1996 to 45.4% in 2011 [8]. The AIDS Concern conducted a survey from 2016 to 2017 and also reported that only 16%, 22%, and 29% of young women who had sex with their boyfriends, regular sex partners, and irregular sex partners, respectively, used condoms every time during sex in the past 6 months [9]. The WHO reported a positive relationship between unsafe sex practices and the incidence of STIs [10]. Hence, the burden caused by STIs is avoidable by minimizing unsafe sex, such as having consistent condom use.

A few systematic reviews have demonstrated that mobile health (mHealth) interventions through interactive instant messaging (IM) are effective for improving health behaviors, for example, smoking cessation [11], medication adherence [12], weight management [13], and blood pressure control [14,15]. In the sexual health arena, to the best of our knowledge, no study has yet evaluated the effectiveness of a personalized and brief intervention delivered through IM for enhancing condom use knowledge and attitude, leading to the primary prevention of STIs. In addition, from our experience in evaluating an interactive computer-based intervention in female university students by a multisite randomized controlled trial, the study implementation led us to understand that a specific, personalized, and tailored intervention to cater to sexual activity recommendations and relationship problems is needed [16]. In the Chinese cultural context of pervasive sexual conservatism, it is unclear whether an IM-delivered brief intervention to promote condom use would be acceptable and welcomed by university students. Therefore, the research question of this study is whether an IM-delivered brief intervention by nurses is feasible to educate about safer sex practice among sexually active university students in Hong Kong.

The study aims to investigate the feasibility of an IM-delivered brief intervention to promote safer sex practices among sexually active university students in Hong Kong. The objectives of the study are to design a nurse-led brief intervention based on Information, Motivation, and Behavioral skills (IMB) model of Fisher and Fisher [17] and to investigate the feasibility of an IM intervention for improving safer sex practices using the feasibility framework reported by Bowen et al [18].

## Methods

### Study Design

The CONSORT (Consolidated Standards of Reporting Trials) eHealth checklist was followed as a reporting guideline to report and appraise the IM-based intervention [19]. Implementation and results of the web-based questionnaire were reported according to the Checklist for Reporting Results of Internet E-Surveys (CHERRIES) checklist [20]. The feasibility study was conducted from August to October 2021.

### Recruitment

**Figure 1:**
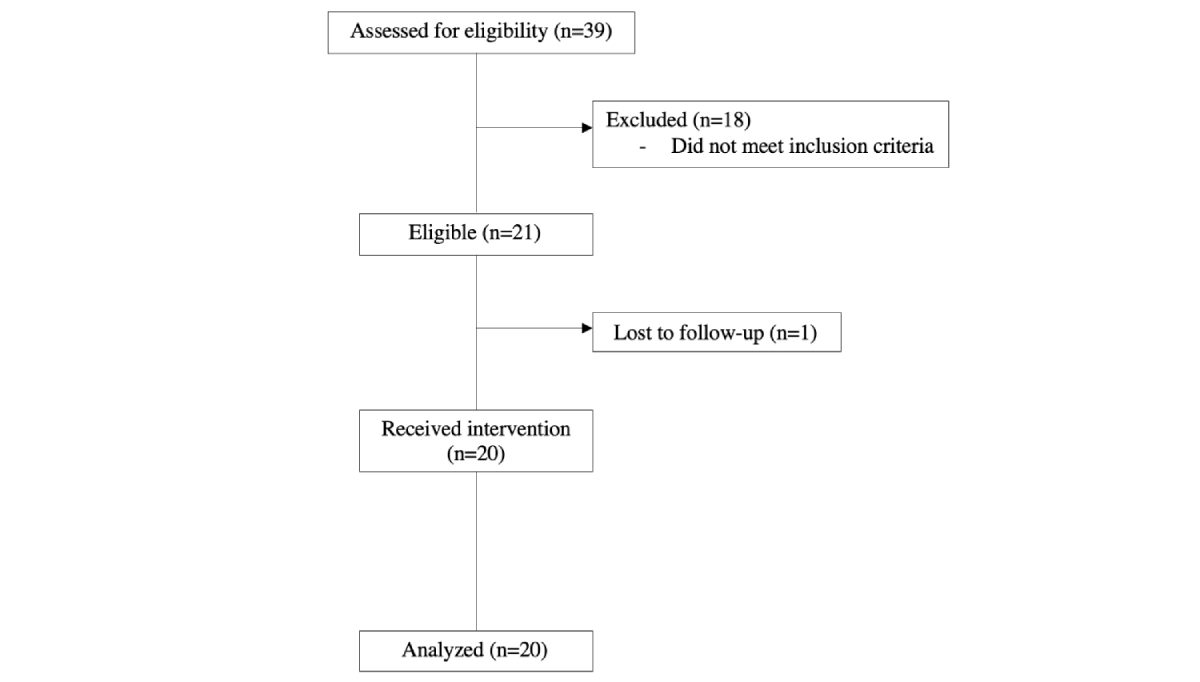
The flow of participants through the study.

Subjects were recruited through snowball sampling at local universities between September and November 2021. An electronic flyer was sent through IM (ie, WhatsApp; Meta Platforms, Inc) to inform potential participants about the purpose of the study, participation eligibility, time commitment, and incentives for study participation. The inclusion criteria were being an emerging Chinese adult aged between 18 and 29 years, being able to read Chinese, having a smartphone with WhatsApp installed, and having had sexual activities in the past 12 months. The exclusion criteria were being pregnant, having a psychiatric illness, and having received information related to contraceptives and STIs from universities, hospitals, clinics, or nongovernmental organizations in the past 12 months. The sample selection process is shown in Figure 1. Eligible participants were asked to sign a web-based consent form for study enrollment and were invited to provide 3 available time slots for receiving the 30-minute nurse-led brief intervention.

### Nurse-Led IM Intervention

#### Intervention Development

The development of the intervention was done by our team, who are experts in the field of sexual health and the development of IM-based interventions. Our nurse-led brief intervention was designed based on the Fisher and Fisher [17] IMB model. A systematic review found that brief interventions designed according to the IMB model were successful in increasing condom use [21]. The model consists of 3 constructs that influence behavioral adherence: that are information, motivation, and behavioral skills [17]. A total of 3 goals were developed accordingly: (1) provide information about STIs and condom use; (2) motivate participants to use condoms with motivational interviewing techniques; and (3) facilitate behavioral skill acquisition to increase condom use, lubricant application, and their purchase.

Regarding the intervention content, we addressed favorable and unfavorable views, including condom use reliability and effectiveness; the pleasure or excitement of condom use; the stigma of condom use; the embarrassment of condom negotiation; condom purchasing; and sexual coercion prevention related to condom use.

#### Implementation Process

A registered nurse who is an expert in sexual health promotion oversaw the intervention delivery throughout the study. The IM conversations were first initiated by providing personalized feedback on the participant’s consistency in condom use, as reported on the baseline survey. Second, further IM engagement explored their reasons for condom use and emphasized their decision to protect themselves by using a condom for the prevention of STIs and pregnancy. Third, the nurse identified the underlying reasons for their consistent and inconsistent condom use and then provided personalized advice, myth clarification, and further motivated participants by emphasizing the importance of condom use at every sex, even with a stable partner. Fourth, continued IM exchanges provided a menu of strategies and conversation techniques to use in condom use negotiations or in verbalizing delaying sex whenever unwanted. Finally, further IM prompts provided support on final tips about safe sex and enhancing self-efficacy for condom use, lubricant use, and its purchase. Overall, the IM messages were phrased in a way to communicate a sense of optimism to use condoms for safety and comfort in the dating relationship. It was conducted in an empathetic, respectful, and nonjudgmental manner. The whole real-time conversation lasted for around 30 minutes through WhatsApp. They were reminded to text the nurse again for any inquiries. A standardized guideline was developed as a reference for the nurse to derive the intervention under the IMB model (Multimedia Appendix 1).

IM was considered for intervention delivery because of its real-time, private, and persuasive nature [22]. IM allows real-time interactions, the use of stickers and emojis, and the delivery of URL links as resources. Also, the personalization of IM conversations is suitable to the needs of those participants, who are passive and sensitive when it comes to sex education. In addition, we provided flexibility for participants to receive the intervention at the most suitable moment; that the intervention time was scheduled after the completion of the baseline questionnaire.

Apart from the 30-minute brief intervention conversations, a booster dose of 5 daily messages was also provided 2 weeks after the brief intervention delivery to further reinforce safer sex knowledge and positive sexual health attitudes. An example of an interaction between the nurse and a participant on WhatsApp is provided in Multimedia Appendix 2.

In order to protect the privacy and confidentiality of the participants during the IM intervention, the messages should not reveal personal identification or other sensitive information, such as HIV status. Only the principal investigator and personnel for data analysis were permitted to access the raw data and study content. All the downloaded messages were stored with passcode protection and will be destroyed after a set period.

#### Data Collection

A baseline questionnaire containing the number of sexual intercourses, the number of protected sexual intercourses in the past 12 months, and the Multidimensional Condom Attitudes Scale (MCAS) was given on the entry of the study (T0). Another questionnaire with the same set of measurements at baseline and questions evaluating acceptability, demand, implementation, practicality, integration, and limited efficacy was distributed 1 week after the completion of the IM intervention (T1). The first author downloaded all the WhatsApp chat data for feasibility evaluation. Incentives of US $18 cash were given for compensating participants for their time and efforts in the study evaluation.

#### Demographic Data

Individual characteristics such as age, sexual orientation, relationship status, and gender of the sex partner were obtained at baseline. A history of childhood sexual abuse experience and sexual health were also asked at baseline to ensure personalized conversation and advice could be made during the intervention. The telephone numbers of the participants were collected to facilitate the delivery of the IM intervention.

#### Feasibility Evaluation Criteria

The feasibility of the brief intervention was investigated according to the recommendations of Bowen et al’s [18] feasibility framework, which includes the focal areas of (1) acceptability, (2) demand, (3) implementation, (4) practicality, (5) integration, and (6) limited efficacy.

##### Acceptability

We examined participants’ perceived appropriateness and satisfaction level with the intervention. Questions included “What is your satisfaction with the intervention on a scale of 1-10?” “Do you think the intervention is appropriate for young adults aged between 18 and 29 years?” “Do you find it embarrassing when discussing sexual health issues during the intervention?” “Do you have any disagreement with the contents?” and “Whether you would recommend the intervention to friends?” Participants could also voice out recommendations for improvement.

##### Demand

We examined the actual use of the intervention, which was reflected in the number of subjects who consented to join the study and its retention rate. All the text messages downloaded were also reviewed for content classification. Participants’ views on their interest in and intention to use were also considered. Questions included “Have you ever asked any questions via the IM app? If yes, is the information useful for you?” and “Will you use the intervention again in the future?”

##### Implementation

We investigated the extent of the intervention that can be successfully delivered to the participants. The number of messages delivered and received was measured. We would be interested to know if the information is appropriate to be understood. Questions such as “Do you have more understanding of condom use?” and “Have you tried to communicate with a sexual partner about condom use?” were asked. In addition, participants’ thoughts on using the intervention through an IM app were also asked to obtain qualitative information. For example, “What do you remember the most?”

##### Practicality

We examined the extent to which the intervention could be carried out by using existing resources. The total number of hours for the whole intervention, including the booster dose, was calculated to estimate the resources needed for implementation. In addition, participants were asked to choose the most effective means of promoting sexual health information to emerging adults.

##### Integration

We investigated the extent to which participants were using the intervention and whether the intervention could be integrated into the current system. The perceived fit with infrastructure, perceived sustainability, and costs to the organization and policy bodies were considered. Questions included “Will you use the intervention again if you come up with any questions about sexual health?” and “What do you think about using IM intervention for sexual health promotion?”

##### Limited Efficacy

We examined whether the intervention could result in a potentially promising outcome and its intended effects. The outcome evaluation was conducted by assessing (1) consistency in condom use and (2) condom use knowledge and attitudes. We compared both outcomes by measuring them before and after the intervention.

Consistency in condom use was measured by the percentage of unprotected sexual intercourse with all partners in the past month. Condom use knowledge and attitudes were measured by the 25-item MCAS. A previous study has shown acceptable validity and reliability in the Chinese population [23]. The Cronbach α of the study was >.7 [23]. It contained 5 domains, including the reliability and effectiveness of condoms, the sexual pleasure associated with condom use, the stigma associated with people proposing or using condoms, embarrassment about negotiating and using condoms, and embarrassment about purchasing condoms [24]. The items were answered on a 7-point Likert-scale, with a total score ranging from 7 to 175. The higher the score, the more positive were knowledge, attitude, norms, and self-efficacy related to condom use.

### Data Analysis

Descriptive statistics were used to determine the participants’ characteristics. Due to the small sample size of the study, a quantile-quantile plot was used to assess the normality of the distribution. A 2-tailed paired sample *t* test was done to compare pre- and postintervention performances for outcome measures with a normal distribution, while the Wilcoxon signed rank test was used for outcome measures without a normal distribution. The content of all the downloaded messages was screened by the first author, and important data were noted. Another member of the team counterchecked all content extracted from WhatsApp to ensure treatment fidelity [25]. Statistical analysis was conducted using SPSS Statistics (IBM Corp).

### Ethical Considerations

The study was approved by the Institutional Review Board of the University of Hong Kong/Hospital Authority Hong Kong West Cluster (UW-20-299). Web-based informed consent forms were sent and completed by eligible participants. All messages collected should not reveal personal identification or other sensitive information to protect the privacy and confidentiality of the participants during the IM intervention.

## Results

### Demographics

Among the 21 participants who completed the baseline questionnaire, 20 (retention rate=95%) of them completed the T0 and T1 questionnaires. The age of the participants ranged from 18 to 25 years, with a mean age of 22 years. All the participants were university students. In terms of sexual orientation, 16/21 (76%) were heterosexual, and 5/21 (23%) were either bisexual or homosexual. The participants’ characteristics can be seen in Table 1.

**Table 1:** Characteristics of the participants (n=21).

Characteristics		Value
Age (years), mean (SD)		22 (2.02)
**Sex, n (%)**		
	Male	4 (19)
	Female	17 (81)
**Sexual orientation, n (%)**		
	Heterosexual	16 (76)
	Bisexual or homosexual	5 (24)
**Relationships, n (%)**		
	Single	3 (14)
	In a relationship	13 (62)
	Cohabitation	5 (24)
**Sex of sexual partner, n (%)**		
	Male	16 (76)
	Female	5 (24)
**Sexual health history, mean (SD)**		
	Number of sex partners in the past 3 months	1.2 (0.95)
	Number of times sex happened in the past 3 months	9.9 (8.16)
	Number of condoms used in the past 3 months	6.45 (7.07)
**History of childhood sexual coercion, n (%)**		
	Yes	1 (5)
	No	20 (95)
**History of pregnancy, n (%)**		
	Yes	0 (0)
	No	21 (100)
**Diagnosed with STIs^a^, n (%)**		
	Yes	0 (0)
	No	21 (100)
**Ever had an STI test or pap smear test, n (%)**		
	Yes	1 (5)
	No	20 (9)

^a^STI: sexually transmitted infection.

### Feasibility Evaluation Criteria

#### Acceptability

Among the 20 participants, the level of satisfaction was high (mean 9.10, SD 1.25). All of them considered that the intervention was appropriate for emerging adults aged between 18 and 29 years. A total of 19/20 (95%) participants did not feel embarrassed when discussing sexual health issues during the intervention. Participants claimed that through the use of emoji and stickers in WhatsApp, they were able to express their emotions and opinions dynamically, which made them feel more comfortable and casual during the chatting session. No participants disagreed with the content in the chat sessions, and all participants would recommend the intervention to friends. Some of the participants also provided practical recommendations. First, participants suggested the provision of more information on the myths about condom and contraceptive use, condom use decision-making and communication with partners, and the availability of condoms in Hong Kong. In fact, during the booster dose, we received messages to ask further questions related to condom use, STIs, and HPV vaccination, which could be included in the content of future interventions. Second, it was suggested to have a mutual sharing personal sexual health information between participants and the nurse. For instance, sharing of personal experiences and problems encountered in dating relationships or during sexual intercourses. The nurse can further ask about individual sexual activities, preferences, and habits. These could make the participants more relatable to the nurse during the intervention and enhance personalized information.

#### Demand

During the recruitment, 39 potential participants were approached, and 21 consented after screening for eligibility and willingness to join the study, with a response rate of 54%. Around 20 of them were retained in the intervention and completed the posttest questionnaire (ie, retention rate of 95%), with only 1 participant lost to follow-up. Among all downloaded messages, the chat session content covered condom use, STIs, HPV vaccination, relationship issues, same-sex activities, refusal of sexual activities, and women’s health checkups. All of them found the message content useful and would use it in the future.

#### Implementation

The chat sessions sent 75 instant messages to each participant on average and received 41 messages from each participant on average with content on lubricant use, contraceptives, STIs, HPV vaccination, and refusal of sexual activities. All participants had a greater understanding of condom use, as reflected in the significant improvement in the MCAS scores. Additionally, 19/20 (95%) tried to communicate with their partners about the use of condoms after the intervention. For qualitative information, we asked participants about the most memorable content. Participants indicated that they particularly remembered the messages about condom use (8/20, 40%) and STIs (7/20, 35%).

#### Practicality

In this study, we had 1 nurse manage all chat sessions, including the 30-minute brief intervention, and 5 daily messages as a booster dose 2 weeks after the brief intervention. The total number of hours spent was around 10 hours. A participant reflected that the response speed during the chat session varied, which might be due to the fact that a nurse had to handle 2 participants at the same time. Nevertheless, 13/20 (65%) of the participants suggested that smartphone apps are an effective way to promote sexual health information, while all of them indicated that mass media (ie, Instagram and Facebook) could be an effective way for promotion.

#### Integration

All participants indicated that they would use the IM intervention again if they came up with any sexual health questions in the future. Participants also claimed that mobile phones are a convenient and comfortable way to receive information about sexual health issues.

#### Limited Efficacy

Due to the small sample size of the study, a quantile-quantile plot was used to assess whether the data set followed a normal distribution (Multimedia Appendix 3). The primary outcome of MCAS showed a normal distribution, and the results of the pre- and posttest are shown in Table 2. The results of the paired-samples *t* test showed a significant increase in the total mean score of condoms use knowledge and attitudes measured by MCAS from 124 (SD 10.32) to 133 (SD 11.90; *t_19_*=3.727; *P*=.001). The Cohen effect size was large (*d*=.81). The mean increase in the MCAS score was 9.05 (95% CI 3.97-14.13). A total of 3 MCAS subscales showed significant results. There was a mean increase in reliability and effectiveness of condoms (*P*=.003), sexual pleasure associated with condom use (*P*<.001), and embarrassment about purchasing condoms (*P*=.03). On the contrary, stigma associated with people proposing or using condoms and embarrassment about negotiating and using condom were not significant.

**Table 2:** The change on condom use knowledge and attitudes by Multidimensional Condom Attitudes Scale (MCAS).

Outcomes	Pretest, mean (SD)	Posttest, mean (SD)	*t* test (*df*)	95% CI	*P* value
Condom use knowledge and attitudes (total MCAS score)	124.05 (2.31)	133.10 (2.66)	3.73 (19)	3.97 to 14.13	.001
Reliability and effectiveness of condoms	26.85 (1.01)	29.55 (1.01)	3.38 (19)	1.03 to 4.37	.003
Sexual pleasure associated with condom use	17.80 (0.76)	21.05 (0.76)	4.56 (19)	1.76 to 4.74	<.001
Stigma associated with people proposing or using condoms	31.30 (0.83)	32.05 (0.83)	0.66 (19)	–1.62 to 3.12	.52
Embarrassment about negotiating and using of condoms	29.10 (1.06)	29.05 (1.06)	–0.06 (19)	–1.81 to 1.71	.95
Embarrassment about purchasing condoms	19.00 (1.80)	21.40 (1.80)	2.42 (19)	0.32 to 4.48	.03

On the other hand, condom use consistency did not show a normal distribution in the normality testing. Therefore, nonparametric testing could be a better representation of the results. The Wilcoxon signed rank test was used to compare the condom use consistency before and after the IM intervention. The increase in the mean score of the condom use consistency was not significant (*Z*=–1.41; *P*=.16), indicating that the intervention was not effective in improving condom use consistency in the study.

## Discussion

### Principal Findings

This study demonstrated that a nurse-led IM-delivered brief intervention is feasible, content-relevant, and acceptable to sexually active Chinese emerging adults for promoting sexual health. In addition, this study demonstrated preliminary efficacy in promoting greater condom knowledge and more favorable attitudes about condoms. These findings are encouraging since, according to the IMB model, improvements in knowledge and attitude are important precursors to behavior changes [17].

This study is the first to use smartphone IM apps for delivering sex education in Hong Kong. The rapid rise in smartphone use provides an opportunity to use such mHealth technology for important health promotion interventions. The IM-delivered intervention has been reported to be highly acceptable in young people aged between 16 and 29 years in Australia, as it is more personal and informal [26]. It can also engage a larger number of individuals personally for a low cost [26]. Another qualitative study, targeting young people aged between 16 and 24 years in the United Kingdom, found consistent results that the delivery of knowledge through IM is friendly and convenient [27]. The findings are in line with our data that the nurse-delivered text messages were able to engage this particular age group regarding sexual health topics in a comfortable and private manner.

Although there are findings that suggest potential benefits of such interventions, many mHealth interventions were found to have low certainty in the evidence [28]. Also, studies evaluating IM-delivered intervention for improving contraceptive-related outcomes have had mixed results when implemented for primary prevention [29]. A total of 2 studies provided inconsiderable effects on sexual health outcomes, such as the percentage of protected sex acts and use of effective contraception, after the delivery of the IM-delivered intervention [30,31]. Another 2 studies showed significant improvements in both reproductive health knowledge and the odds of using condoms in young adults after receiving the IM-delivered intervention [32,33]. Differences in the results could be due to the design and duration of the intervention. In terms of the intervention design, a 2-way interactive component can be more effective in promoting sexual health outcomes [33]. In terms of the intervention duration, it can range from weeks to months [27]. A study suggested that the participants identified redundancies when receiving messages over a long intervention period (ie, 2 years) [30]. Around 20% of the participants in another study in Australia found the 12-weekly messages annoying [26]. Hence, future research on evaluating the IM-delivered interventions in terms of appropriateness of design, optimal intervention length, message delivery frequency, and effectiveness in promoting sexual health knowledge and safe sex practice is necessary.

Our results were analyzed using Bowen et al’s [18] systematic feasibility framework. Condom use knowledge and attitudes were significantly increased. In addition, the results regarding the acceptability of the intervention were perceived to be appropriate according to participants’ needs. Participants were satisfied with the intervention and content. We achieved a retention rate of 95.2%. Most participants showed interest in the intervention, with an average satisfactory score of 9. Also, all of them would recommend the intervention to others. Furthermore, the intervention did not create embarrassment in the discussion due to its private nature. With reflection on the implementation process and results of this feasibility study, we recognized that (1) the IM-delivered intervention was feasible and created no embarrassment during discussions, (2) discussing sexual health issues directly helped to clarify myths, (3) screening for condom use consistency and sexual orientation helped tailor conversations, (4) condom use inconsistency was primarily due to the displeasure of using a condom and alternative contraceptives being used for pregnancy prevention, which also suggested a lack of awareness of STI prevention, and (5) most conversations could use such gender-neutral terms as “they or them” and “partner,” except for discussions of condom or protective barrier practical use tips to fit both heterosexual and lesbian, gay, bisexual, transgender, queer, plus (LGBTQ+) relationships, and (6) using WhatsApp stickers in a conversation helped to create a comfortable atmosphere and establish rapport to facilitate further discussions. This study highlighted the usefulness of using Bowen et al’s [18] framework for systematically evaluating the feasibility of an innovation.

The booster dose stimulated participants’ interest in asking further questions about sexual health-related issues. The booster can not only provide new information to participants but also deliver similar intervention message content to refresh participants’ memories. This helps maintain behavioral changes over time [34]. Previous studies containing booster interventions indicated that participants were able to revisit the content and reinforce their knowledge in these sessions [21,35]. In our booster session, diverse topics such as sexual needs, frequency of sexual activities, delay of sexual intercourse, and STIs were covered. Some participants also raised personal and private questions related to STIs during the booster session. This may indicate that participants needed time to digest the information received initially, which fostered the trust and courage needed to further ask personal questions. Therefore, booster intervention is essential for some participants to assimilate information and share their information at their own pace [27].

### Implications

Sex education is essential as a primary prevention strategy for emerging adults due to the underestimation of the risk of unprotected sex [9]. A survey from AIDS Concern found that nearly 40% of surveyed young women underestimated their risk of STIs, while 80% of them were assessed as the high-risk population [9]. Although Hong Kong has a relatively low level of youth sexual activity, recent studies have brought up concerns about the inadequacies of sex education in Hong Kong [36]. Conventional sex education programs or school-based sex education are some common means of promoting condom use in Hong Kong, however, they are still not mandatory. In addition, the teaching hours are largely insufficient. Half of the schools in Hong Kong offered 5 hours or less of sex education in a school year [37]. The conservative culture on sexual health issues also shaped Hong Kong people’s views and attitudes [36]. Although Hong Kong is an internationalized city that accepts cultural diversity and open-mindedness, the general public is still reserved and implicit when it comes to sex education [36]. This study supports the idea that an IM-delivered intervention is potentially effective in filling the gap due to its personalized and proactive delivery mode.

Further efforts should be made to scale up the implementation of the intervention in the community. In total, 1494 messages were sent and received between the nurse and 20 participants during the 30-minute brief intervention and a 5-day booster dose of daily messages 2 weeks later. Also, a participant reflected that the response speed during the chat session varied. The reduced instantaneity may affect the intervention effect. Compared with face-to-face talk and counseling, this intervention was delivered with mHealth technology through free smartphone apps, which were more cost-effective in terms of delivery. We did not conduct a cost-effectiveness analysis in this feasibility study. Previous studies have been done to evaluate the cost-effectiveness of IM interventions targeting smoking cessation [38] and weight management [39]. Smartphone-based interventions using SMS text messaging or IM have demonstrated good cost-effectiveness due to their low cost and scalability. However, a cost-effectiveness assessment on this specific topic has not been done. The main concern of a nurse-led IM intervention could be the requirement of more manpower if it is conducted on a large scale. Having said that, we would suggest developing an intervention manual and training a group of sexual health counselors for implementation efficiency at a large scale. In addition, with the advancement of technology, the training manual can be used for the further development of chatbots, which can assist in intervention delivery automatically. A systematic review reported a significant effect of a chatbot intervention in reducing depression [40]. While a randomized controlled trial also found chatbot intervention to be effective in promoting fertility awareness and preconception health [41]. An intervention protocol with standard answers should be well-designed to maintain a high standard of IM chat session content.

### Strengths and Limitations

The strength of the study was in providing a realistic assumption on the feasibility of an IM-delivered brief intervention on promoting safer sex practices in the community. Our evaluation was conducted systematically based on Bowen et al’s [18] framework. The intervention was able to create a friendly, comfortable, nonjudgemental atmosphere that allowed participants to think and ask any questions about sexual health. The participants were also able to gain knowledge and more favorable attitudes about condom use.

The limitation of this study was the small sample size, which might affect the generalizability of the results. In addition, a comparison group was not established, so caution is needed when interpreting its findings. However, it is appropriate to have a small sample size to determine whether the intervention is feasible before carrying out a large-scale study [18]. Additionally, it could be cost-ineffective to increase the sample size without conducting a feasibility study. Another limitation was the short follow-up period. It is very likely to take longer than 2 weeks postintervention to see changes in behavior [21]. Future research with a longer time frame would be able to more fully assess the outcome of this intervention.

### Conclusion

An IM-delivered brief intervention was feasible and acceptable. The intervention was also demonstrated to be practical and effective in enhancing participants’ general knowledge and attitudes toward condom use. The intervention was able to minimize embarrassment when discussing sensitive topics. Although intervention implementation can be time-consuming and labor-intensive, the study findings informed IM-based sexual health intervention development or modifications to enhance sexual health promotion among sexually active university students. More research is needed to evaluate the effectiveness and cost-effectiveness of such an intervention at a larger scale with trained counselors and evaluate its long-term effects by using a rigorous design, such as a randomized controlled trial.

## Appendix

Multimedia Appendix 1 A guideline for the nurse to derive the intervention under the Information, Motivation, and Behavioral skills model during the intervention.

Multimedia Appendix 2 Examples of the interaction between participants and the nurse during the brief intervention on WhatsApp. Multimedia content (blue, right) and answers from participants (orange, left) are shown.

Multimedia Appendix 3 QQ plots for Multidimensional Condom Attitudes Scale total score and condom use consistency.

## Abbreviations

CHERRIES: Checklist for Reporting Results of Internet E-Surveys
CONSORT: Consolidated Standards of Reporting Trials
HPV: human papillomavirus
IM: instant messaging
IMB: Information, Motivation, and Behavioral skills
LGBTQ+: lesbian, gay, bisexual, transgender, queer, plus
MCAS: Multidimensional Condom Attitudes Scale
mHealth: mobile health
STI: sexually transmitted infection
WHO: World Health Organization
