# Supplementary material for: Nurse-Led Brief Intervention for Enhancing Safe Sex Practice Among Emerging Adults in Hong Kong Using Instant Messaging: Feasibility Study
Source: JMIR Form Res. 2024 Mar 20;8:e52695. doi: 10.2196/52695 (PMC10993122; doi:10.2196/52695)
Supplement: Multimedia Appendix 1 [file formative_v8i1e52695_app1.docx]

**Table S1**. Guideline and content of the adaptive sexual health intervention.

| **IMB component** | **Performance objectives** | **Advice in brief motivational intervention** |
| --- | --- | --- |
| **Information** | 1. Understand reliability and effectiveness of condom | - Discuss myths about not using condoms and provide counterarguments for disadvantages of condom use Explain the importance of consistent condom use to prevent pregnancy, STIs, and HIV |
|  | 1. Understand condom can prevent both STIs and pregnancy | - Identify the risks of getting pregnant and STIs without a condom - Explain the satisfactory effectiveness of condom as a form of contraception - Explain some of the STIs are asymptomatic and the importance of checking body for signs of a sore, blister, rash, or discharge |
| **Motivation** | 1. Sex with a condom can still be pleasurable | - Describe how the lubricant in condom help with overstimulation and make it more enjoyable - Explain that condom add excitement at the foreplay and condom as an erotic part of the foreplay - Encourage to discuss and try different colours, flavours and texture of condoms for fun |
|  | 1. Use condoms every time when having (anal or vaginal) sexual intercourse even with stable sexual partner | - Provide feedback on their condom use consistency - Encourage to discuss personal risk perception related to STIs, HIV/AIDS and pregnancy unless condoms are used consistently - Explain that use of condoms is a sign of trust, respect, and care, which is elements for long term relationship - Explain how the condom can make comfort to manage their own sexual health - Explain mutual monogamy is helpful in preventing STIs but reliability of self-reported sexual history is unknown due to social desirability in Chinese culture. Also, there is confusion between serially monogamous and real monogamy in layman. And honestly of previous partner is unknown |
|  | 1. Obtain/ buy condoms can be easy | - Suggest places where they can obtain condoms for free - Suggest places where they can obtain condoms online for reducing embarrassment, if applicable - Reassure the confidence to deal with embarrassment when buying or obtaining a condom - Suggest how to DIY own dental dams by using a latex condom |
|  | 1. Always take condom along | - Indicate having sex with a one-nightstand or within a steady relationship can both lead to health risks - Perceive self at risk for STIs/pregnancy if failure to carry condoms - Discuss the advantages and disadvantages of taking a condom along - Suggest how long condoms can be carried in wallet or bag without decreasing efficacy |
| **Behavioral skills** | 1. Negotiate condom use with a sexual partner | - Describe their feelings about discussing condom use with a partner - Suggest discussing condom use with partners earlier instead of during romantic situations - List out steps of successful negotiation - Provide 7 conversation techniques to negotiate for condom use - Discuss how to initiate condom use in difficult situations - Check if the partner pressures into having unwanted sex or having sex without condom and provide conversations techniques to stop sex if sexual coercion occurs |
|  | 1. Use condoms and lubricants correctly | - Provide step-by-step ways on condom use (including male, female condoms, and dental dams) - Describe how to put on, take off and dispose of a condom - Provide tips on correct condom use - Explain two types of lubricant and its usage - Remind expiry date of condom and lubricant |
