# Supplementary figures and images for: Nurse-Led Brief Intervention for Enhancing Safe Sex Practice Among Emerging Adults in Hong Kong Using Instant Messaging: Feasibility Study
Source: JMIR Form Res. 2024 Mar 20;8:e52695. doi: 10.2196/52695 (PMC10993122; doi:10.2196/52695)

**Figure S1**s An example of an interaction between the nurse and a participant on WhatsApp.


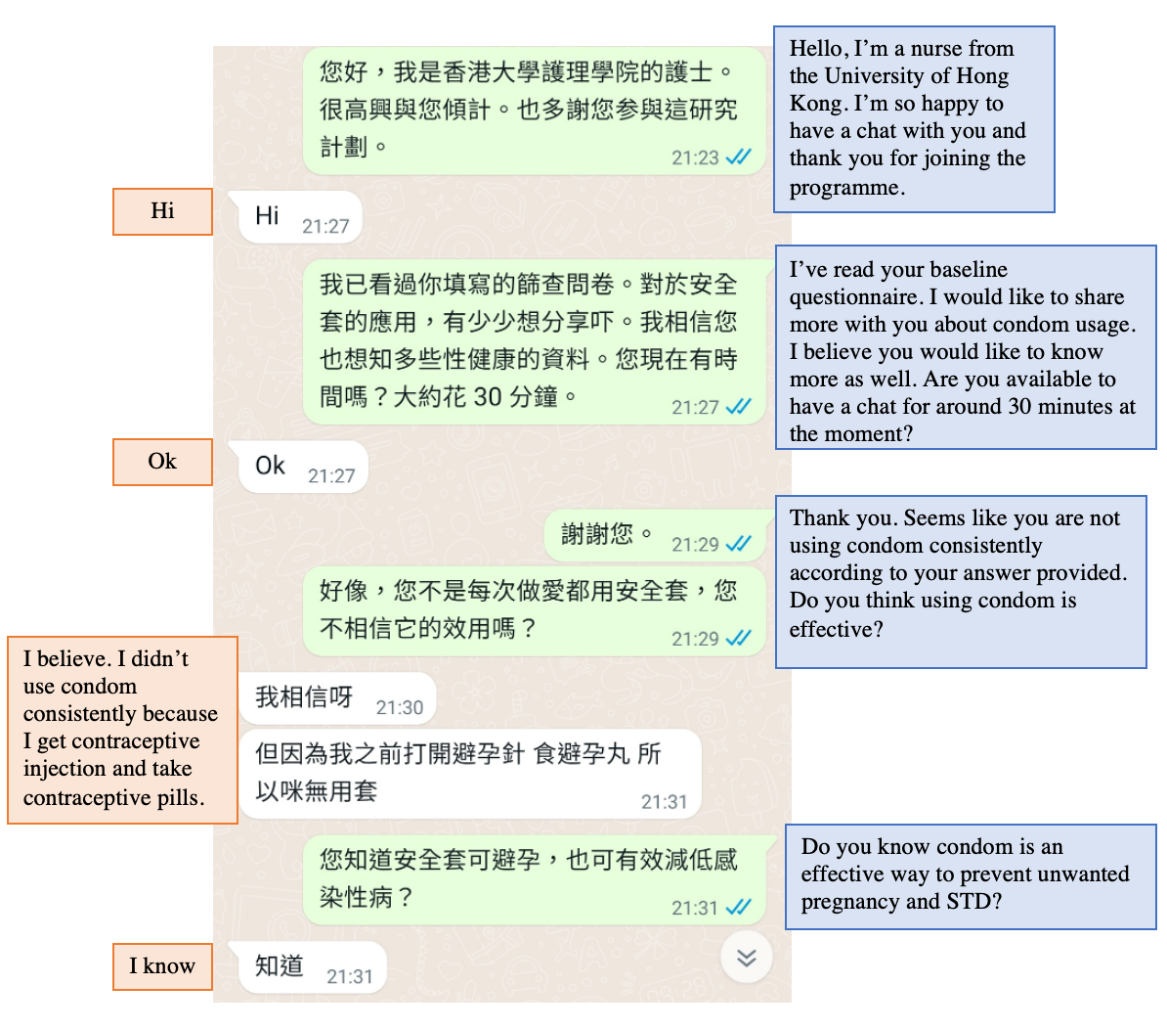

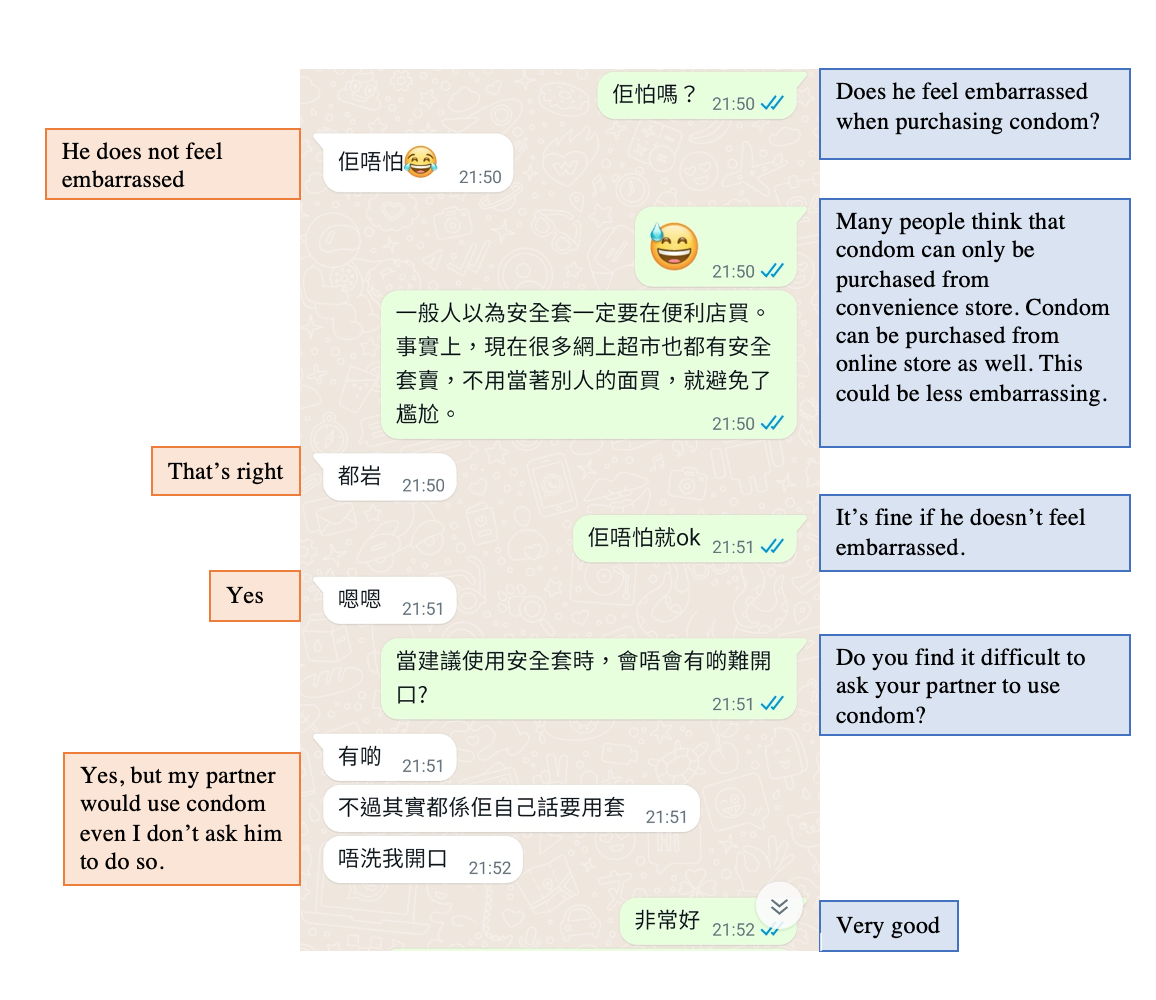

Supplement: Multimedia Appendix 2 [file formative_v8i1e52695_app2.docx]
