# Supplementary material for: Nurse-Led Brief Intervention for Enhancing Safe Sex Practice Among Emerging Adults in Hong Kong Using Instant Messaging: Feasibility Study
Source: JMIR Form Res. 2024 Mar 20;8:e52695. doi: 10.2196/52695 (PMC10993122; doi:10.2196/52695)

**Figure S1**. QQ-plots for MCAS total score and condom use consistency.

**QQ- plot for MCAS total score**


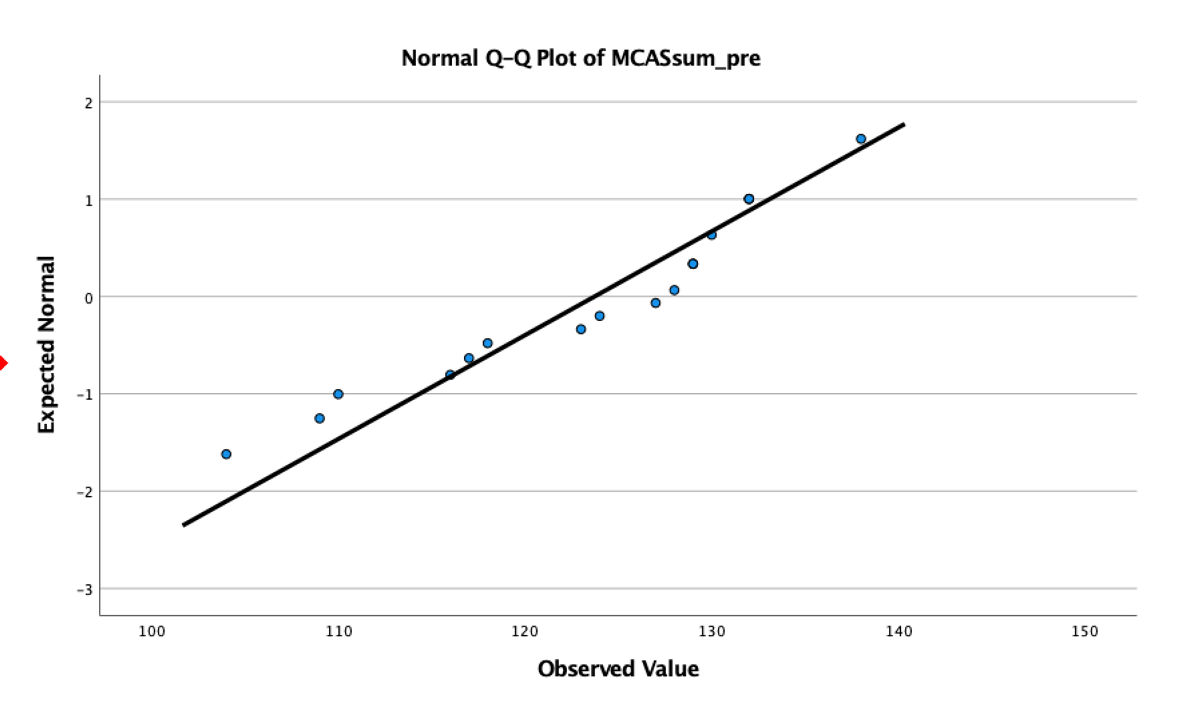


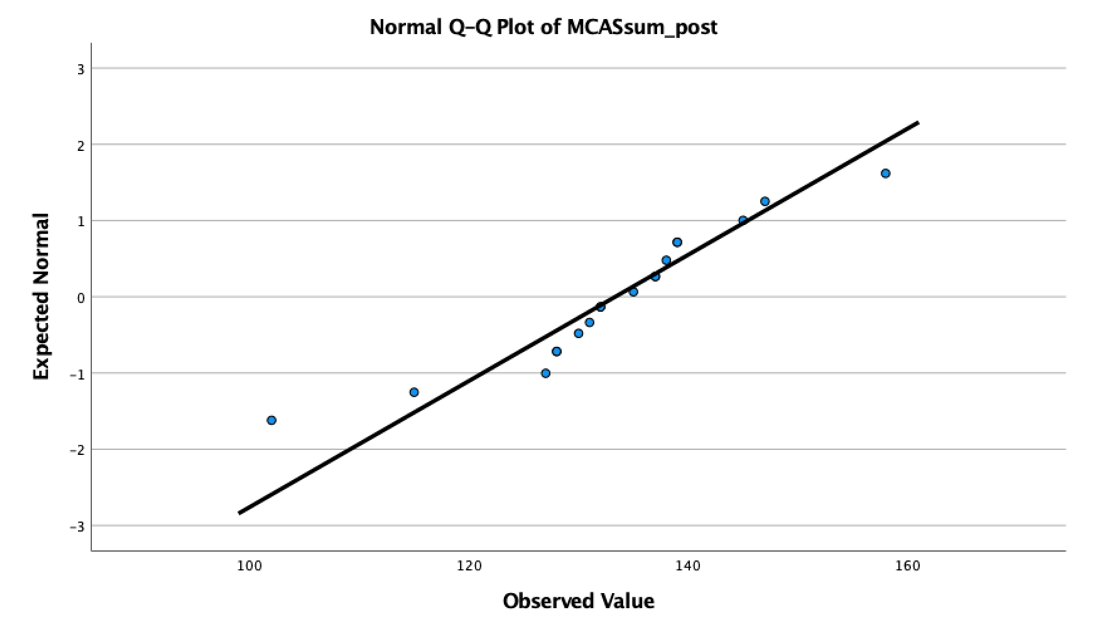


**QQ-plot for condom use consistency**


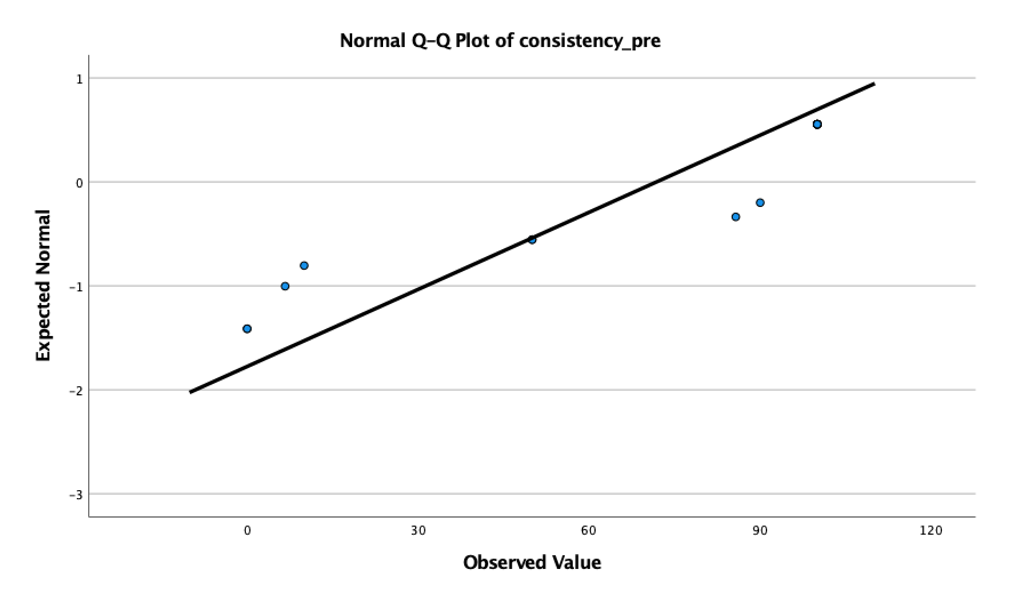


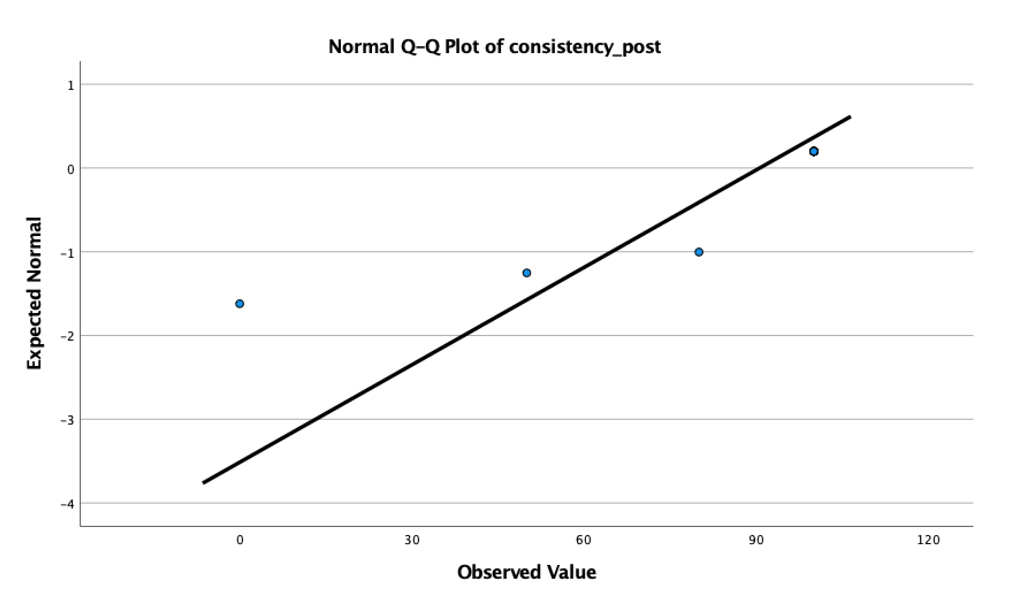

Supplement: Multimedia Appendix 3 [file formative_v8i1e52695_app3.docx]
